# Neutrophil extracellular traps as therapeutics target in vascular aging

**DOI:** 10.3389/fimmu.2025.1657938

**Published:** 2025-10-08

**Authors:** Jie Mao, Shuang Wu, Zelin Yan, Guiping Huang, Yihua Yu

**Affiliations:** ^1^ The Second School of Clinical Medicine, Zhejiang Chinese Medical University, Hangzhou, China; ^2^ Department of Neurology, Zhejiang Hospital, Hangzhou, China; ^3^ The First School of Clinical Medicine, Zhejiang Chinese Medical University, Hangzhou, China; ^4^ General Ward Department, Zhejiang Hospital, Hangzhou, China

**Keywords:** NETs, vascular aging, cardiovascular diseases, ang II, inflammation

## Abstract

Blood vessels are critical in systemic aging with arteries stiffening and calcifying due to chronic inflammation and oxidative stress, driving age-related cardiovascular and cerebrovascular diseases. In this review, neutrophil extracellular traps (NETs) —web-like structures composed of decondensed chromatin, histones, and antimicrobial proteins released by neutrophils—are explored as therapeutic targets in vascular aging. NETs are vital for pathogen defense, but their excessive activation leads to inflammation and vascular pathologies, promoting endothelial dysfunction, inflammatory aging, and vascular remodeling in diseases such as hypertension, atherosclerosis, myocardial infarction, heart failure, atrial fibrillation, ischemic stroke, and Alzheimer’s disease. Increasing evidence supports that modulating NETs through inhibitors or scavengers can reduce inflammatory responses, preserve endothelial integrity, and improve prognosis. As a potential therapeutic target, growing attention has been directed toward exploring the balance between NET induction, inhibition, and degradation.

## Introduction

With the accelerating pace of population aging, the study and focus on aging-related diseases have become increasingly critical. Among these, cardiovascular diseases (CVDs) are one of the most prevalent age-associated conditions, accounting for a significant proportion of morbidity and mortality worldwide (1). Vascular aging constitutes the core pathological basis of CVDs and central nervous system (CNS) disorders. As age advances, structural remodeling of the vascular wall occurs, characterized by elastin fragmentation, increased collagen deposition, vascular calcification, and chronic low-grade inflammation (inflammaging) (2–4). These changes directly result in increased arterial stiffness and endothelial dysfunction. Emerging biomarkers, such as circulating inflammatory factors, epigenetic age, and vascular calcification scores, offer promising tools for the early identification of high-risk individuals (5, 6).

Neutrophils, the most abundant leukocytes in human blood, are rapidly recruited to sites of injury and infection via the vasculature (7). They limit pathogen spread through phagocytosis, degranulation, and NET release (8, 9). However, hyperactivated neutrophils frequently lead to adverse outcomes detrimental to host tissues and immune responses, leading to exacerbated organ damage (10). Of particular interest are neutrophil-released NETs, which promote endothelial injury, oxidative stress, immune dysregulation, and procoagulant states, thereby accelerating arterial stiffness and dysfunction. These alterations directly contribute to inflammatory aging and vascular aging (11–14).

## The structure and function of NETs

NETs are web-like structures released by neutrophils during immune defense processes, primarily composed of decondensed chromatin associated with cytoplasmic and granule proteins, such as myeloperoxidase (MPO) and neutrophil elastase (NE) (15, 16). The formation of NETs, referred to as NETosis, is a specialized form of neutrophil cell death triggered by pathogenic infections, pro-inflammatory stimuli, or damage-associated molecular patterns (DAMPs) (17, 18). This process depends on the coordinated actions of signaling pathways involving protein-arginine deiminase-4 (PAD4), NE, and Toll-like receptor 4 (TLR4) (19, 20), and is accompanied by changes in reactive oxygen species (ROS) levels and the regulation of apoptosis-related signaling pathways (21–23). These mechanisms promote the decondensation of chromatin and histones, allowing chromatin to expand from the nucleus into the cytoplasm. Ultimately, the neutrophil cell membrane ruptures, releasing chromatin and antimicrobial proteins to form the NET structure (24).

Under physiological conditions, NETs play a critical role in host defense by directly capturing and eliminating pathogens and modulating cytokine release to influence local inflammatory responses. This trapping mechanism, facilitated by the web-like structure of NETs, enhances pathogen clearance by concentrating antimicrobial proteins and promoting phagocytosis by other immune cells (25). Additionally, NETs modulate local inflammatory responses by releasing cytokines such as IL-8, which recruit and activate immune cells to coordinate acute infection resolution (26). In certain contexts, NETs may contribute to tissue repair by clearing necrotic debris and supporting wound healing (27). However, aberrant or persistent activation of NETs can lead to chronic inflammation, immune dysregulation, and tissue damage. For instance, in autoimmune diseases such as systemic lupus erythematosus (SLE), impaired clearance of NETs may result in sustained exposure to self-antigens, thereby promoting the production of autoantibodies and the deposition of immune complexes, which exacerbates inflammatory responses (28, 29). Additionally, the role of NETs in the tumor microenvironment has garnered increasing attention. NETs not only facilitate cancer metastasis through the capturing cancer cells but may also enhance tumor cell resistance to chemotherapy (30, 31). The induction of NETs can be achieved through various stimulatory methods, with classic inducers including phorbol 12-myristate 13-acetate (PMA) and lipopolysaccharide (LPS) (32, 33).

## NETs damage vascular endothelial cells and exacerbate vascular dysfunction

In 2024, Professor Mahmoud Abdellatif innovatively proposed eight molecular hallmarks as common markers of cardiovascular aging: autophagy dysfunction, loss of proteostasis, genomic instability, epigenetic alterations, mitochondrial dysfunction, cellular senescence, neurohormonal dysregulation, and inflammation (34). Aging endothelial cells (ECs) are recognized as a primary hallmark of vascular diseases (35). The clearance of senescent cells has been shown to alleviate aging-associated symptoms and extend healthspan (36). Endothelial cell senescence exhibits characteristic features of normal cellular aging, such as irreversible growth arrest, increased expression or activation of p53, upregulation of p21^WAF1/Cip1^ and cell cycle inhibitor p16^Ink4a^, elevated senescence-associated β-galactosidase activity (SA-β-Gal) (37), and the presence of the senescence-associated secretory phenotype (SASP) (38). Endothelial cell senescence often begins with endothelial cell damage, which leads to endothelial dysfunction, induces a pro-inflammatory state, further exacerbates endothelial senescence, and promotes the progression of vascular diseases such as atherosclerosis, hypertension, and stroke (39). Current experiments have demonstrated that NETs have significant toxic effects on Human Umbilical Vein Endothelial Cells (HUVECs). NETs not only promote the proliferation of HUVECs but also activated the nuclear factor-κb (NF-κB) pathway, up-regulated the expression of important inflammatory and angiogenic molecules, including vascular cell adhesion molecule-1 (VCAM-1), intercellular adhesion molecule-1 (ICAM-1), metallopeptidase-14 (MMP-14), vascular endothelial growth factor A (VEGFA), and interleukin-6 (IL-6), and induced the formation of immature neovascularization (40, 41). Another study revealed a significant enrichment of mitophagy- and ferroptosis-related signaling pathways in HUVECs incubated with NETs (42). NETs convert HUVECs into procoagulant and pro-inflammatory phenotypes (43, 44).

Vascular dysfunction is an early hallmark of various age-related diseases. Its core feature is closely linked to endothelial cell dysfunction. As a single layer of cells lining the lumen of blood vessels, endothelial cells serve as the direct interface between blood and the vessel wall (45). Endothelial cells regulate vascular tone by releasing vasodilators such as nitric oxide (NO) and vasoconstrictors such as endothelin-1 (ET-1) in response to changes in blood flow (46). Recent studies have further uncovered the critical role of NETs in endothelial dysfunction and vascular pathological processes (47). In the progression of atherosclerosis, NETs induce endothelial cell damage, promote monocyte adhesion, and drive macrophages toward a pro-inflammatory phenotype, thereby accelerating the formation and instability of atherosclerotic plaques (48). Furthermore, NETs act as scaffolds for platelets and coagulation factors, enhancing the coagulation cascade and inhibiting fibrinolysis, which promotes thrombus formation and stabilization in deep vein thrombosis (DVT) and arterial thrombosis (49, 50). NETs are also closely linked to vascular aging, as they accelerate vascular aging by promoting endothelial damage, immune dysregulation, and a procoagulant state. Inflammaging can enhance NETs formation, while NETs-mediated inflammatory cytokine release and increased oxidative stress further exacerbate vascular dysfunction (51, 52).

## NETs promote age-related diseases

A substantial body of research demonstrates that NETs interact extensively with various proteins and participate in numerous physiological pathways, including inflammation and oxidative stress. This delicate balance underscores the significant role of NETs in growth and development. However, NETs produced during the aging process disrupt vascular function, potentially exacerbating the progression of CVDs and neurodegenerative disorders.

## Cardiovascular disease

**Figure 1:**
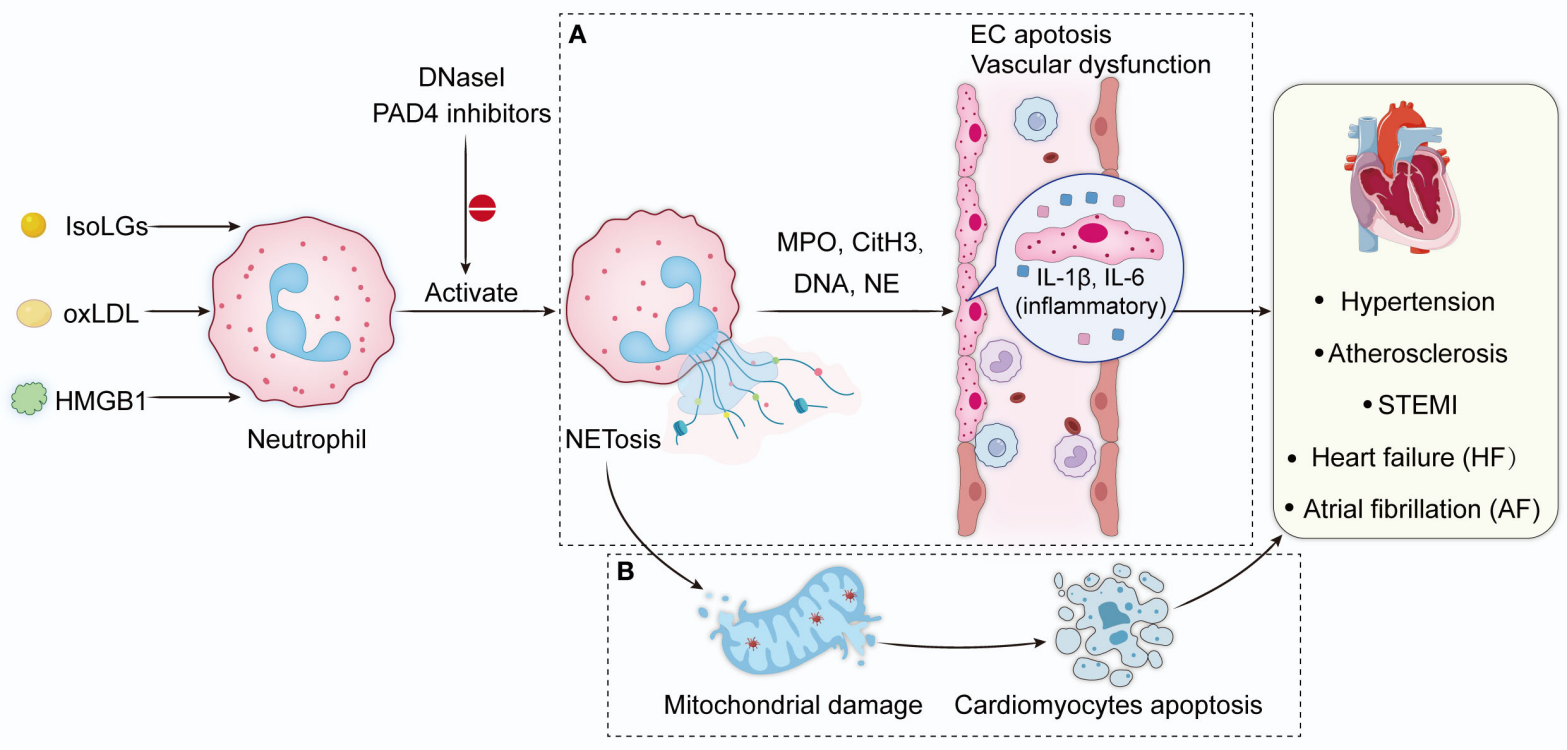
NETs in vascular diseases. Peroxidation products, inflammation directly stimulate neutrophil migration and induce NETs formation. DNase I and PAD4 inhibitor reduce NET formation. **(A)** NETs activate innate immunity by releasing extracellular DNA and histones, and also attack the vascular endothelium by releasing MPO and NE, which further increase oxidative stress and drive the formation of inflammatory mediators IL-6 and IL-1β, and ultimately disrupts the endothelial barrier. **(B)** NETs induce mitochondrial damage in cardiomyocytes, leading to autophagic apoptosis and further cardiac dysfunction. NETs, neutrophil extracellular traps; IsoLGs, Isolevuglandins; HMGB1, high-mobility group box 1; oxLDL, oxidized low-density lipoprotein; DNase I, deoxyribonuclease I; PAD4, Protein arginine deiminase 4; CitH3, citrullinated histone H3; MPO, Myeloperoxidase; NE, neutrophil elastase; IL-6, interleukin-6; IL-1β, interleukin-1β.

In cardiovascular diseases, NETs primarily drive vascular remodeling, thrombosis, and chronic inflammation, amplifying arterial stiffness and cardiac dysfunction common in aging. Increased vascular resistance due to reduced vessel diameter is a key pathophysiological mechanism contributing to hypertension (53). Several signaling pathways are involved in vascular dysfunction and the progression of hypertension, including calcium channels, the nitric oxide–nitric oxide–soluble guanylyl cyclase–cyclic guanosine monophosphate (NO-NOsGC-cGMP) pathway, vascular remodeling pathways, and upstream regulators such as the renin-angiotensin-aldosterone system (RAAS), oxidative stress-related pathways, and immune/inflammatory pathways (53). Changes in intracellular calcium concentration are a key mechanism regulating the contractile state of vascular smooth muscle cells. Treatment of neutrophils with the mechanosensitive calcium channel transient receptor potential vanilloid 4 (TRPV4) agonist increases intracellular calcium and NETosis, both in a dose-dependent manner. Jaya et al. further discovered that citrullinated histones in NETs disrupt endothelial cell integrity, contributing to vascular dysfunction (54). NETs not only activate innate immunity via the release of extracellular DNA and histones but also attack the vascular endothelium through the release of MPO and NE, which further increase oxidative stress and disrupt the endothelial barrier (55). Concurrently, elevated MPO-DNA complexes, as reported by Smith et al., correlate with endothelial apoptosis in hypertensive patients, highlighting NETs’ role in amplifying oxidative stress (56) (**Figure 1**). Isolevuglandins (IsoLGs), products of lipid peroxidation, further stimulate NET formation, with studies showing that the IsoLG scavenger 2-hydroxybenzylamine (2-HOBA) reduces NET accumulation and improves blood pressure in animal models (57). Another point of concern is that NETs, through their interactions with endothelial cells and coagulation factors, directly enhance coagulation activity, inducing a hypercoagulable state and increasing thrombotic risk (58). In patients with primary hypertension and hyperhomocysteinemia (HHcy), studies show that circulating deoxyribonuclease I (DNase I) can effectively degrade NETs, thereby reducing the risk of thrombosis (56). These findings collectively suggest that NETs integrate inflammatory, thrombotic, and oxidative pathways to drive hypertension, with potential therapeutic implications for targeting NETosis to mitigate vascular damage.

Atherosclerosis, as a hallmark of vascular aging, is closely associated with age-related cellular dysfunction (59). NETs exert multifaceted effects across different stages of atherosclerosis. Warnatsch et al. demonstrated that NETs activate proinflammatory immune responses during early plaque formation by releasing DNA and granule protein, driving the production of inflammatory mediators such as interleukin-1β (IL-1β) (60). This process is further exacerbated by oscillating high-fat diets (alternating between high-fat diets and regular diets), which create a more pronounced proinflammatory microenvironment (61). As plaques mature, hypercholesterolemia impairs DNase-mediated NET clearance, allowing persistent NETs accumulation that enlarges the necrotic core, as shown by Döring et al. (62). Concurrently, oxidized low-density lipoprotein (oxLDL) promotes NETosis by increasing intracellular chloride concentrations (63). Notably, histone H2A within NETs promotes monocyte adhesion and amplifies inflammatory dissemination under endotoxemic conditions, accelerating plaque progression (64). In experimental models of recurrent ischemic stroke associated with atherosclerosis, circulating cell-free DNA (cfDNA) was found to induce NETosis and activate the absent in melanoma 2 (AIM2) inflammasome within atherosclerotic plaques, thereby exacerbating inflammation and destabilizing the plaques (65). Researchers at Harvard Medical School demonstrated that the delivery of PAD4 inhibitors via collagen IV-targeted nanoparticles effectively reduces NET formation, preserves endothelial integrity, and prevents plaque erosion (66). These studies collectively underscore NETs as a central mediator of chronic inflammation and plaque destabilization in atherosclerosis, highlighting their potential as a therapeutic target to alleviate vascular aging.

These thrombotic and inflammatory contributions in atherosclerosis and hypertension parallel the acute injury and remodeling effects of NETs in myocardial infarction, where they amplify local damage and long-term cardiac consequences. NETs accumulate at the culprit site and correlate with infarct size in ST-segment elevation myocardial infarction (STEMI) (67). NETs enhance local thrombosis and inflammation and directly promote fibrotic remodeling through the activation of fibrocytes at the infarct site (68). This dual effect makes them critical mediators of both initial myocardial injury and long-term cardiac dysfunction following STEMI. Reducing neutrophil infiltration and NETs formation attenuates myocardial ischemia/reperfusion injury (I/R) (69, 70). Interestingly, NETs released after tissue injury in myocardial infarction (MI) fundamentally disrupt humoral immunity by inducing widespread lymphocyte death and immunoglobulin loss, leading to impaired mucosal defenses and higher risk of infection (71). However, another study found that NETs in MI also act as modulators of post-infarction inflammation and tissue repair, promoting better cardiac healing and function in MI, especially when pathological thrombosis is not a dominant feature (72). Pharmacological or genetic strategies to inhibit NET formation—for example, PAD4 knockdown, inhibitors such as DNase1, the leukotriene C4 (LTC4) receptor antagonist pranlukast—reduce myocardial injury and improve cardiac function after MI, highlighting the translational potential of targeted NETs in MI therapy (73).

NETs exacerbate chronic cardiac dysfunction in heart failure by promoting inflammation, mitochondrial damage, and pathological remodeling. Elevated NET formation, triggered by inflammatory signals and oxidative stress, amplifies myocardial injury (74–76). For instance, Zhang et al. showed that high-mobility group box 1 (HMGB1) promotes neutrophil recruitment and NETosis in a heart failure with preserved ejection fraction (HFpEF) mouse model, exacerbating diastolic dysfunction. Suppression of the HMGB1-NET axis using sodium-glucose cotransporter 2 (SGLT2) inhibitors such as empagliflozin successfully improves diastolic function (77). In the pathophysiological process of heart failure, NET levels are closely associated with disease severity, with NET-induced mitochondrial damage in cardiomyocytes serving as a critical factor. Professor Bo Yu’s team identified a novel VWF-SLC44A2-NET axis involved in the progression of heart failure and demonstrated that elevated NET levels impair mitochondrial function and cardiac performance via the NE-TLR4-PGC-1α pathway (78). Additionally, low-density neutrophils (LDNs) in acutely decompensated HFpEF patients exhibit heightened NET-forming capacity, intensifying chronic inflammation. This suggests that LDNs may serve as a key source of NET formation (79). Studies using a pressure overload model show that the deficiency of developmental endothelial locus-1 (DEL-1) promotes neutrophil infiltration and NET formation via activation of the p38 signaling pathway, leading to myocardial remodeling and dysfunction (80). DEL-1 exerts its protective effects by inhibiting P38 signaling and suppressing NET formation, highlighting its potential as a therapeutic target. Clinical studies show that the dynamic changes in NET markers before and after left ventricular assist device (LVAD) implantation further highlight the close relationship between NETs, inflammation, and thrombosis. The significant post-surgical decrease in NET levels may be associated with the alleviation of inflammatory responses (81).

Similar to heart failure, the formation and functional mechanisms of NETs in atrial fibrillation (AF) are closely associated with inflammation, fibrosis, and thrombosis development. In patients with AF, NET levels are significantly elevated and positively correlate with abnormalities in left atrial hemodynamics, the grade of spontaneous echocardiographic contrast (SEC), and a prothrombotic state (82). NETs activate fibrosis-related signaling pathways, such as Smad and MAPK, promoting the production of extracellular matrix (ECM) proteins, including collagen and fibronectin, thereby driving fibrosis progression. Additionally, NETs facilitate fibroblast differentiation into myofibroblasts, which exacerbates atrial structural remodeling in AF patients (83, 84). NETs also induce autophagic apoptosis and mitochondrial membrane potential depolarization in cardiomyocytes, resulting in cardiomyocyte atrophy, increased perinuclear granules, and further impairment of mitochondrial function (83, 85). AF induces NET formation predominantly in the left atrial appendage, potentially contributing to the development of left atrial mural thrombi (86). Specific markers of NETosis may serve as prognostic indicators for adverse events in AF, offering novel insights and potential clinical applications for predicting thrombotic risk and optimizing the CHA_2_DS_2_-VASc score (87).

## Cerebrovascular diseases

**Figure 2:**
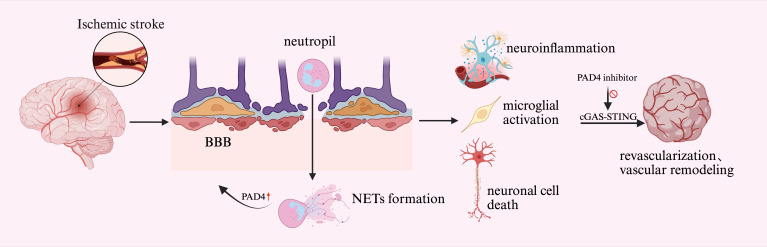
NETs mediated BBB disruption. After ischemic stroke reperfusion, neutrophils enter the brain through the increased permeability of the BBB and release NETs. Overexpression of PAD4 exacerbates BBB ​​disruption. This process triggers microglia activation, ultimately leading to neuronal cell death. Next, NETs further impair hemodialysis and vascular remodeling after brain injury through the cGAS-STING pathway. PAD4 inhibitors mitigate brain damage by inhibiting this pathway. BBB, blood-brain barrier; PAD4, Protein arginine deiminase 4; cGAS-STING, cyclic GMP-AMP synthase-stimulator of interferon genes.

In cerebrovascular diseases, NETs exacerbate neurovascular unit dysfunction, including blood-brain barrier disruption and neuronal damage, contributing to both acute and chronic aging-related pathologies. NETs play a detrimental role in various stages of ischemic stroke. During the acute phase, platelet-derived HMGB1 mediates NET formation, worsening stroke outcome by promoting local thrombosis and inflammation (50, 88). Notably, MPO inhibitors stabilize atherosclerotic plaques, reduce the incidence and severity of vascular occlusion, limit inflammation-driven tissue damage, and prevent adverse cardiovascular events (89). A common phenomenon in stroke is the destruction of the blood-brain barrier (BBB). The BBB is a specialized vascular structure formed by endothelial cells that separates the CNS from the peripheral circulation, and plays a crucial role in regulating molecular pathways in and out of the brain, and protecting neural tissues from pathogens (90, 91). After reperfusion in ischemic stroke, partial recovery of the BBB leads to increased permeability, allowing neutrophils to enter the brain by altering the tight junctions of endothelial cells (92). During the delayed phase, a significant influx of peripheral neutrophils migrates to the affected brain tissue, where they release NETs (**Figure 2**). This process exacerbates BBB disruption, triggers microglial activation, and ultimately contributes to neuronal cell death (93). Researchers have demonstrated that overexpression of PAD4 exacerbates BBB breakdown and reduces revascularization in an ischemic mouse model (94). These findings indicate NETs impair revascularization and vascular remodeling after stroke. NETs contribute to cerebrovascular complications via cyclic guanosine monophosphate-adenosine monophosphate synthase-stimulator of interferon gene(cGAS-STING) activation and type 1 interferon response in the ischemic brain (95). A team has developed a self-assembled liposomal nanocarriers loaded with a PAD4 inhibitor to inhibit NETosis and reduce downstream inflammatory factors by inhibiting the cGAS-STING pathway to ameliorate brain injury in mice with ischemic stroke (96). Similarly, another team achieved neuroprotection by targeting neutrophils to deliver NETs inhibitors to the site of brain injury, thereby reducing neuroinflammation and oxidative damage (97). This strategy may provide a foundation for the development of stroke-targeted therapeutic diagnostics.

Alzheimer’s disease (AD) is a gradual, progressive neurodegenerative disease characterized by the accumulation of neuritic plaques of β -amyloid (amyloid-β, Aβ) in neuronal cytosol outside of neurons and the accumulation of abnormally hyperphosphorylated tau proteins within neurons (98, 99). Neutrophil accumulation has been reported at amyloid plaques (100). NETs are also detected in both the blood vessels and parenchyma of AD model mice and in patients with AD. These NETs are often located near Aβ plaques (101). NETs components such as MPO, citrullinated histone H3 (CitH3), NE and PAD4 are significantly elevated in the prefrontal cortex of AD model mice. These elevations are associated with increased levels of inflammatory cytokines including tumor necrosis factor-α (TNF-α), IL-1β, IL-6, and interferon-γ (IFN-γ) (102). NETs are pro-inflammatory and cytotoxic, and their release is associated with increased vascular permeability and tissue destruction (103). The formation of NETs contributes to BBB disruption and neuronal damage in AD (104). Strategies that block neutrophil trafficking or directly inhibit NETs reduce the formation of NETs, improve cognitive function, decrease microgliosis, and lower Aβ and phospho-tau pathology in AD model mice (101, 105). These findings suggest that NETs are not only biomarkers of inflammation but active contributors to the progression of AD, driving neurodegeneration and impaired cognition.

## Angiotensin II and NETs synergistically induce vascular senescence

In recent years, the interplay between NETs and the renin-angiotensin system (RAS) has garnered significant attention. Angiotensin II (Ang II), a key effector molecule of the RAS, not only directly induces NETosis but also indirectly facilitates NET release in complex pathophysiological settings via inflammation and oxidative stress. Moreover, NETs, by promoting the release of inflammatory cytokines and ROS, further activate the renin-angiotensin system, establishing a positive feedback loop that may play a pivotal role in vascular aging and the progression of related diseases.

A growing body of evidence reveals the pathophysiological role of Ang II in cardiovascular diseases, including inflammation, metabolic dysfunction, and aging (106). Ang II induces oxidative stress in neutrophils via its Ang II type 1 receptor (AT1R), stimulating the excessive production of ROS and further amplifying this process through the activation of nicotinamide adenine dinucleotide phosphate (NADPH) oxidase. This ROS-mediated oxidative stress directly activates PAD4, leading to histone citrullination and triggering NET release (107). In an abdominal aortic aneurysm (AAA) model Ang II promotes extensive NET formation by inducing DNA extrusion through signaling pathways such as p38 MAPK and PI3K (108). In contrast, researchers at West China Hospital reported that Ang II does not directly activate NETs; rather, when combined with other stimulatory factors such as PMA, the NET-releasing ability of neutrophils was significantly enhanced, accompanied by an increase in AKT phosphorylation and elevated autophagy levels (83).

In addition to direct pathways, Ang II indirectly promotes NET formation by enhancing neutrophil activation and the inflammatory microenvironment. For instance, Ang II significantly activates neutrophils and increases their propensity to release NETs by downregulating Krüppel-like factor 2 (KLF2) within these cells (109). At Ang II-induced lesion sites, the aggregation of NETs not only causes endothelial cell damage but also leads to microthrombosis, resulting in impaired blood perfusion, myocardial ischemia, and capillary rarefaction. This effects ultimately exacerbating downstream organ fibrosis and functional decline (110).

**Figure 3:**
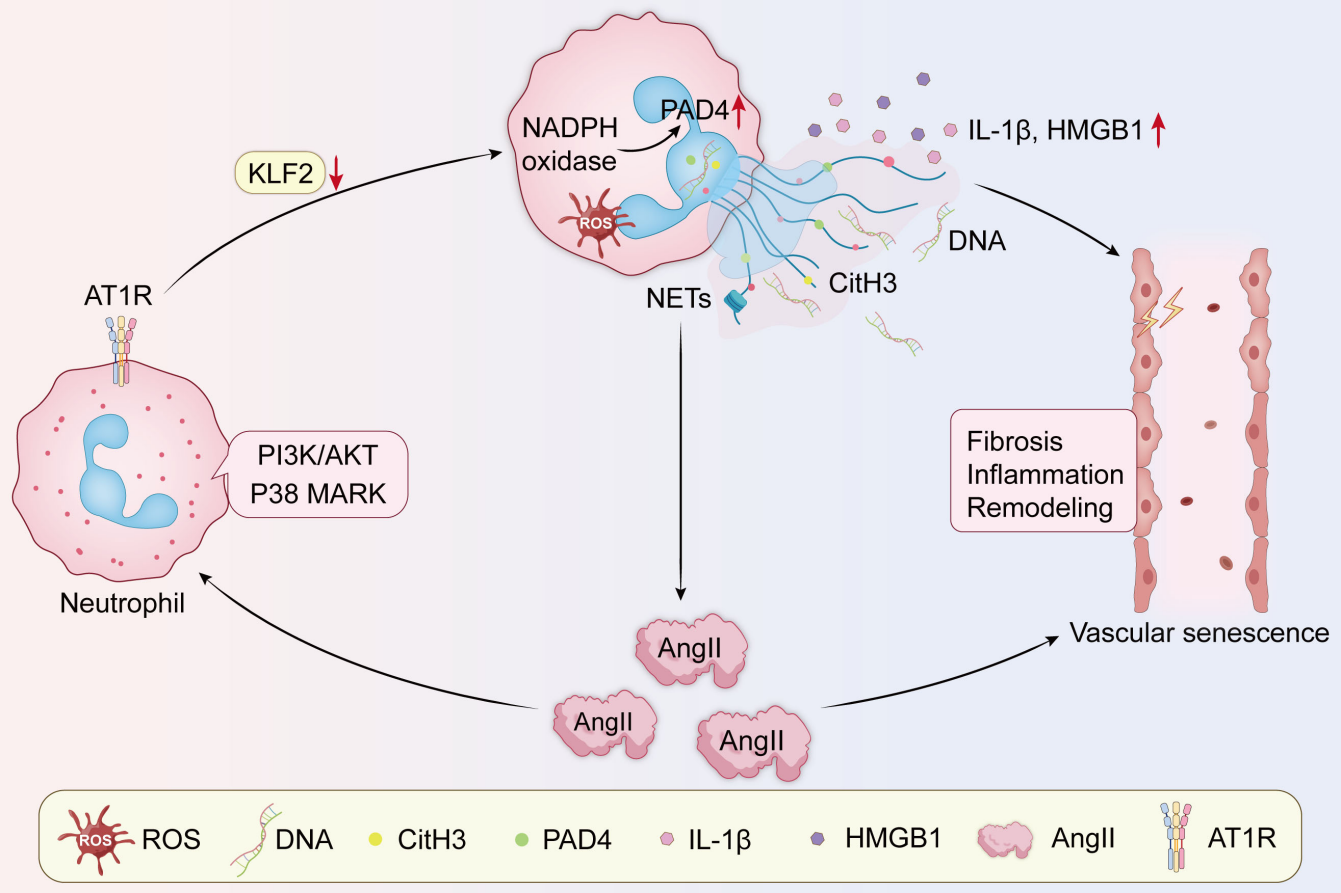
The positive feedback loops between NETs and angiotensin. Ang II acts directly on AT1R, activating NADPH oxidase, upregulating ROS and PAD4 levels, and downregulating KLF2, while activating p38 MARK and PI3K/AKT signaling pathways, promoting DNA efflux to form NETs, which release the pro-inflammatory factors IL-1β and HMGB1, creating a local inflammatory environment and enhancing Ang II expression, resulting in the pathological features of inflammation, vascular remodeling, and fibrosis. All of these may lead to vascular senescence. AT1R, Ang II type 1 receptor; NAPDH, nicotinamide adenine dinucleotide phosphate; ROS, reactive oxygen species; KLF2, Krüppel-like factor 2; IL-1β, interleukin-1β; HMGB1, high-mobility group box 1.

The formation of NETs is not only a result of Ang II and RAS activation but also enhances RAS activity by promoting inflammatory responses and oxidative stress, creating a positive feedback loop (**Figure 3**). This feedback mechanism may play a critical role in vascular aging and the progression of related diseases. The release of substantial amounts of ROS and inflammatory factors (such as IL-1β and HMGB1) during NET formation significantly amplifies RAS activation (83, 110). For instance, in AF studies, NET-associated markers such as CitH3 and cfDNA exhibit a positive correlation with RAS activation. Notably, Ang II further promotes NET formation, resulting in cardiomyocyte damage and mitochondrial DNA (mtDNA) release, with these damage-associated signals driving NET generation via a positive feedback mechanism, thereby exacerbating fibrosis and structural remodeling in AF (83). Components released during NET formation (MPO and DNA) accelerate vascular pathology by inhibiting the PI3K/AKT signaling pathway in smooth muscle cells (SMCs), leading to smooth muscle ferroptosis, exacerbated inflammation, and further increase in Ang II levels within the lesion site (108). This positive feedback mechanism may serve as a critical driving force behind vascular aging and vascular- associated diseases.

## Conclusions and future perspectives

The review synthesizes the multifaced role of NETs in vascular aging and related disease. This discussion revolves around the dual nature of NETs in physiological and pathological contexts. From a physiological perspective, NETs hold significant importance in immune defense by capturing pathogens and facilitating their clearance. However, over-activation drives a shift toward pathological drivers such as chronic inflammaging, oxidative stress, endothelial dysfunction and thrombosis.

NETs exacerbate vascular diseases by promoting vascular remodeling, hypercoagulability, and neuroinflammtion, often amplified through positive feedback loops with Ang II. The review also elucidates the interaction between NETs and vascular diseases. Therapeutically, targeting NET formation (e.g, PAD4 inhibitors) and degradation (e.g, DNase) or upstream modulators (e.g., SGLT2 inhibitors, IsoLG scavengers) holds promise for mitigating these effects and improving outcomes in aging populations.

Unresolved questions include the functional differences in NETs across age groups and populations; for instance, while elderly individuals produce more NETs, their function and value-added stimulation of cells may diminish (111). Controversies persist regarding NETs’ context-dependent roles—beneficial in controlled inflammation versus detrimental in chronic states—and the precise mechanisms linking NET overactivation to vascular senescence. Future research should prioritize elucidating molecular pathways of NET-vascular interactions, developing NET-based biomarkers for early risk stratification and personalized medicine, and conducting *in vitro*/*in vivo* studies to validate population-specific effects. These efforts could advance NET-targeted therapies, ultimately reducing the burden of age-related vascular diseases and enhancing healthspan.
